# Supplementary figures and images for: DAF-16/FoxO and DAF-12/VDR control cellular plasticity both cell-autonomously and via interorgan signaling
Source: PLoS Biol. 2021 Apr 23;19(4):e3001204. doi: 10.1371/journal.pbio.3001204 (PMC8099054; doi:10.1371/journal.pbio.3001204)

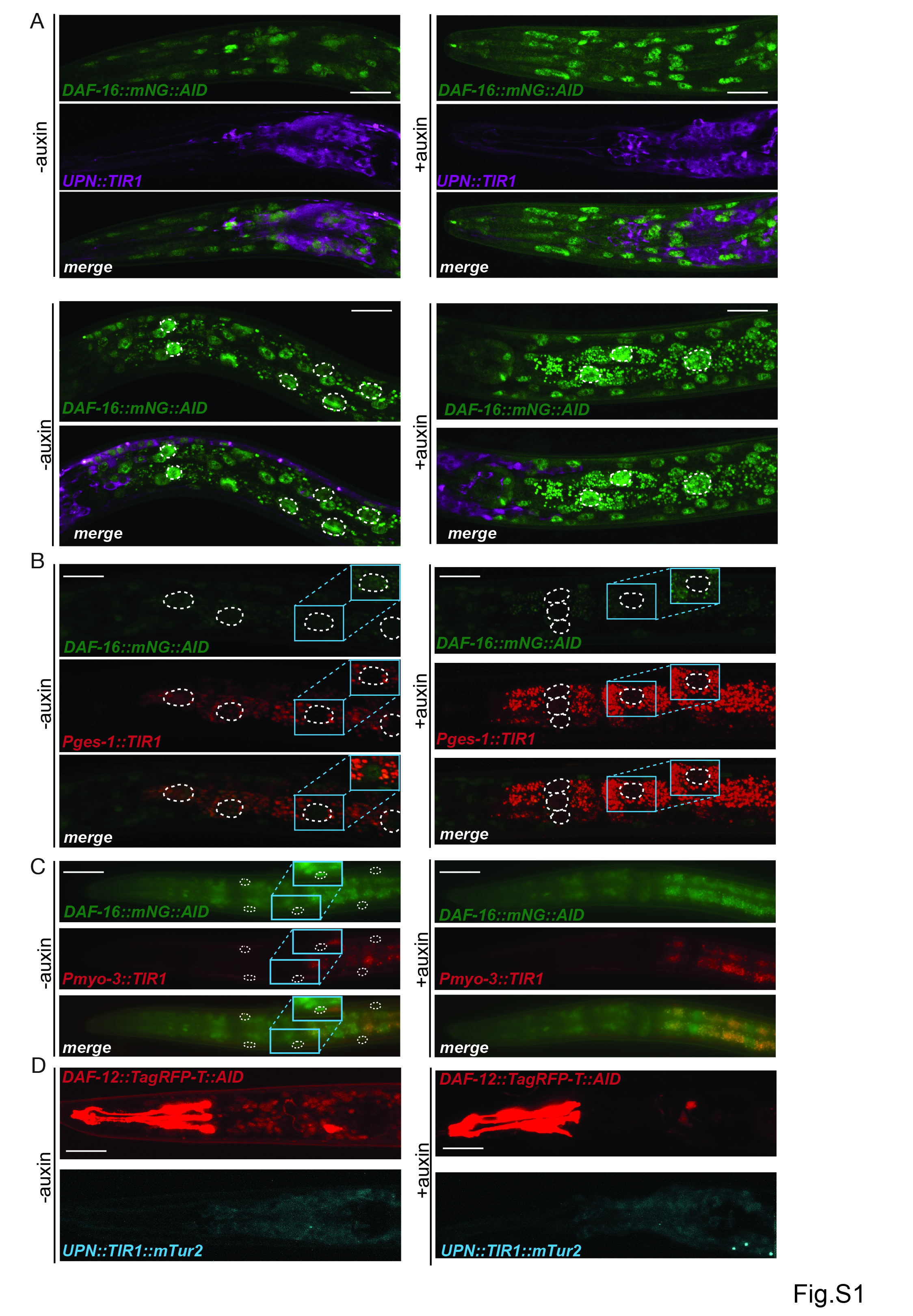

Supplement: S1 Fig — (A) Confocal images of dauers with panneuronal depletion of DAF-16/FoxO under control and auxin treatment conditions. Scale bar, 20 μm. (B) Confocal images of dauers with intestinal depletion of DAF-16/FoxO under control and auxin treatment conditions. Insets show an example of an intestinal nucleus, magnified and enhanced to boost fluorescence signal (both in control and auxin-treated conditions). Scale bar, 20 μm. (C) Epifluorescent images of dauers with body wall muscle depletion of DAF-16/FoxO under control and auxin treatment conditions. Insets show an example of a muscle nucleus, magnified and enhanced to boost fluorescence signal. Scale bar, 20 μm. (D) Confocal images of dauers with panneuronal depletion of DAF-12/VDR under control and auxin treatment conditions. Pharyngeal expression is from a co-injection marker inx-6prom 18::TagRFP. Scale bar, 20 μm. TF, transcription factor. (TIF) [file pbio.3001204.s001.tif]

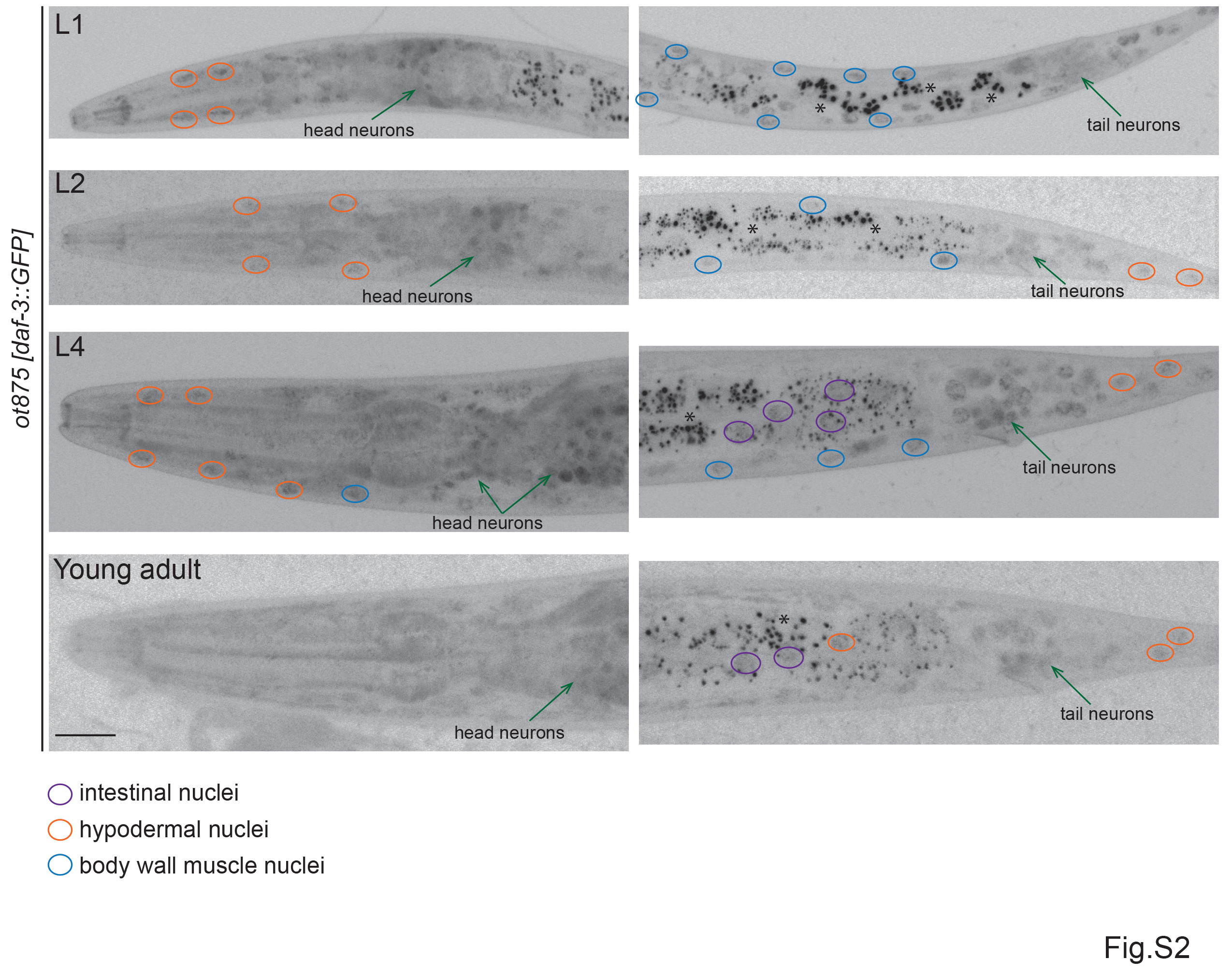

Supplement: S2 Fig — Expression of the daf-3::GFP CRISPR allele at different stages in development in starved conditions. Anterior is to the left on all images. Scale bar, 20 μm (same for all images). (TIF) [file pbio.3001204.s002.tif]

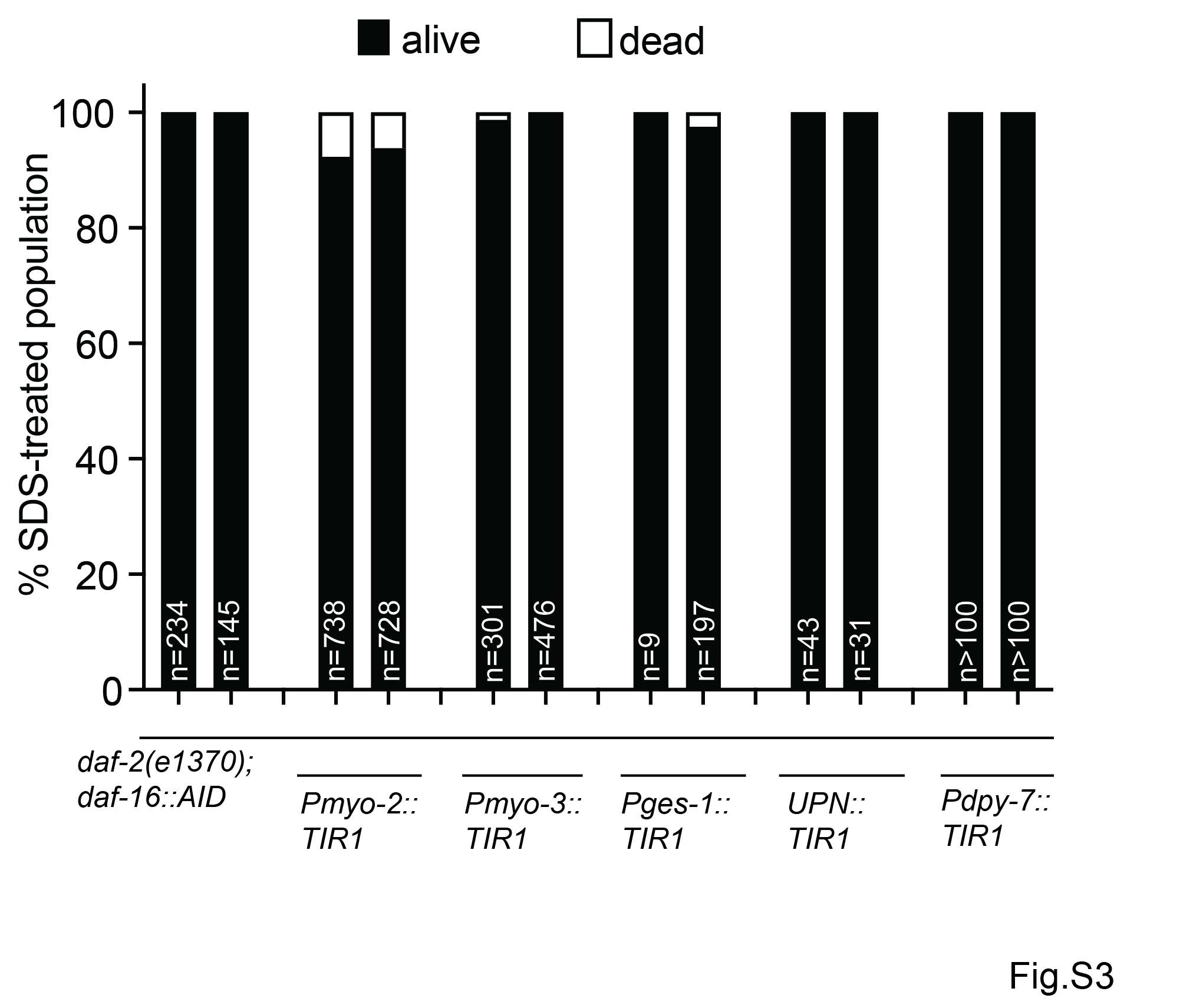

Supplement: S3 Fig — Worms were washed off NGM plates (control and auxin-treated) 3 days after rearing at 25°C and incubated in 1% (m/v) solution of SDS for 30 minutes, with continuous gentle shaking. After washing with water and M9 buffer, the worms were plated on fresh plates and scored as alive if moving. The data underlying this figure can be found in S1 Data. NGM, Nematode Growth Medium. (TIF) [file pbio.3001204.s003.tif]

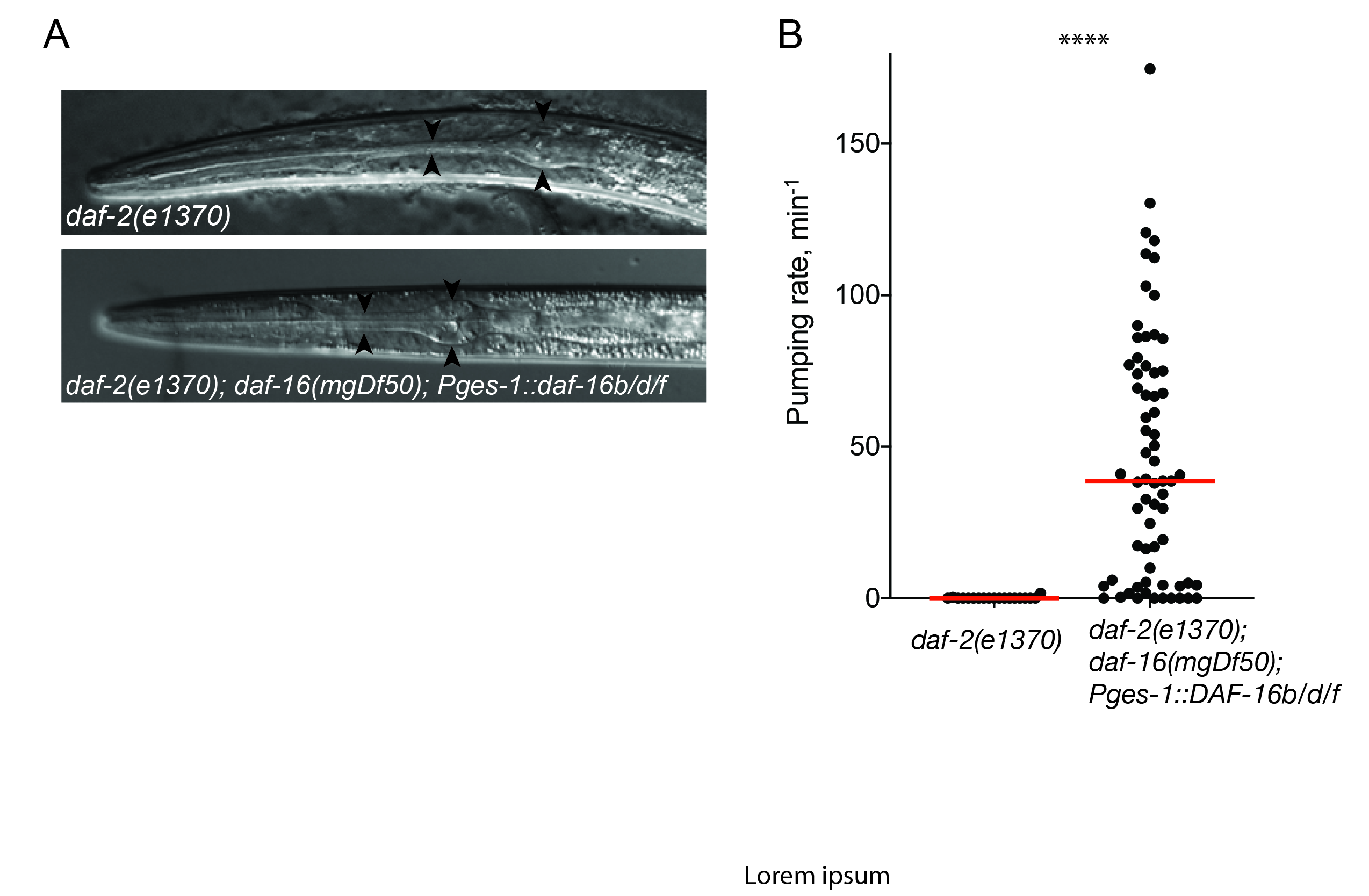

Supplement: S4 Fig — (A) DIC images of a daf-2(e1370) dauer and a dauer-like larva with intestinal rescue of daf-16b/d/f isoforms in the daf-2(e1370); daf-16(Df50) background. Note the deviation from the wild-type filariform pharynx morphology in the latter case. (B) Quantification of pharyngeal activity of the strains depicted in (A). The data underlying this figure can be found in S1 Data. (TIF) [file pbio.3001204.s004.tif]

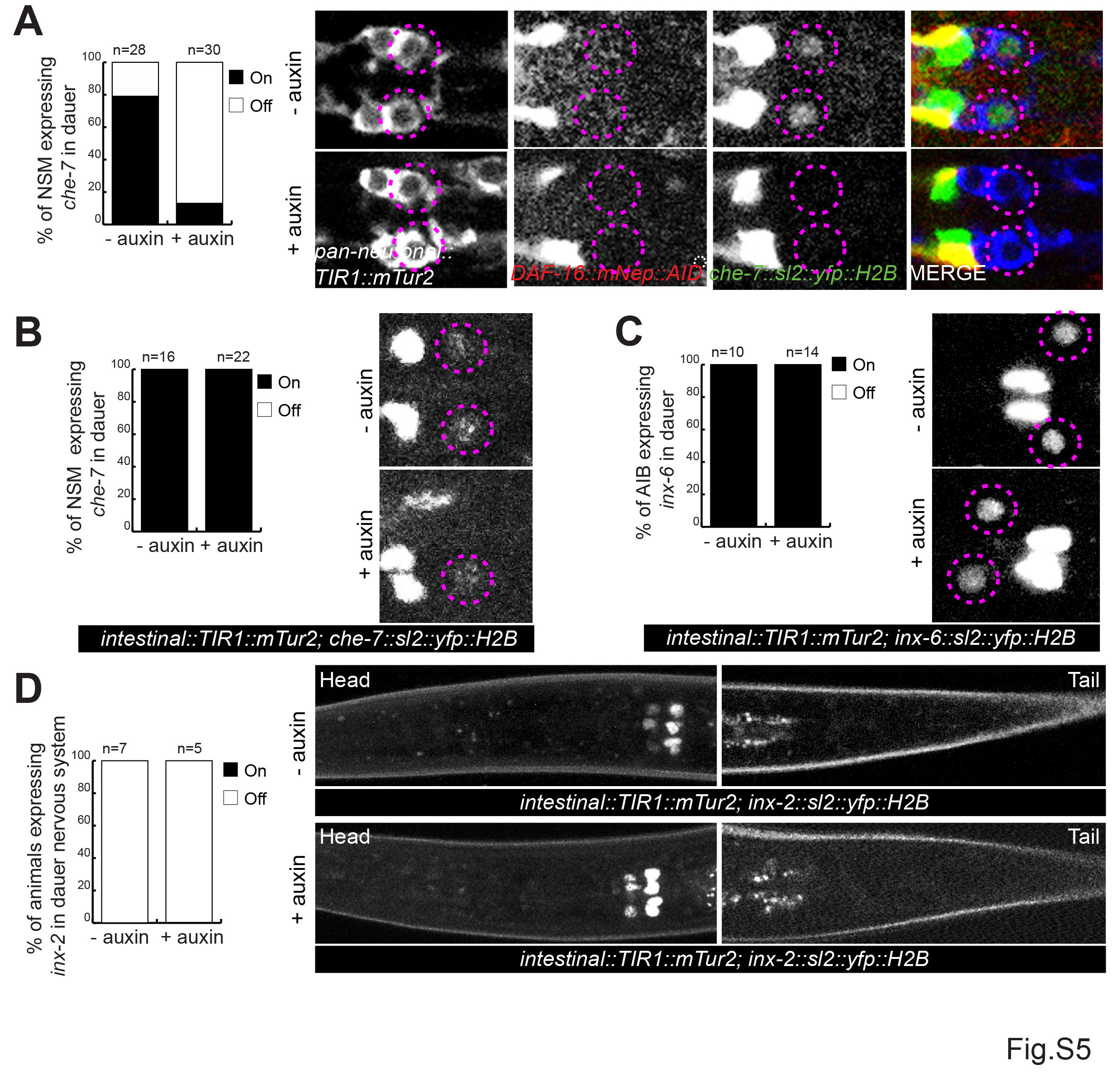

Supplement: S5 Fig — (A) A che-7 reporter (otEx7112) expression is gained in NSM neurons in dauer. This dauer-specific che-7 expression is lost upon panneuronal depletion of DAF-16/FoxO in auxin-treated dauers. (B) Dauer-specific che-7 expression in NSM is unaffected upon intestinal depletion of DAF-16/FoxO in auxin-treated dauers. (C) Expression of an inx-6 reporter allele (ot804) is gained in AIB neurons in dauer. This dauer-specific inx-6 expression in AIB neurons is unaffected upon intestinal depletion of daf-16/FoxO in auxin-treated dauers. (D) Expression of an inx-2 reporter allele (ot906) is down-regulated in multiple neurons in dauer. This dauer-specific down-regulation of inx-6 expression is unaffected upon intestinal depletion of DAF-16/FoxO in auxin-treated dauers. The data underlying this figure can be found in S1 Data. (TIF) [file pbio.3001204.s005.tif]

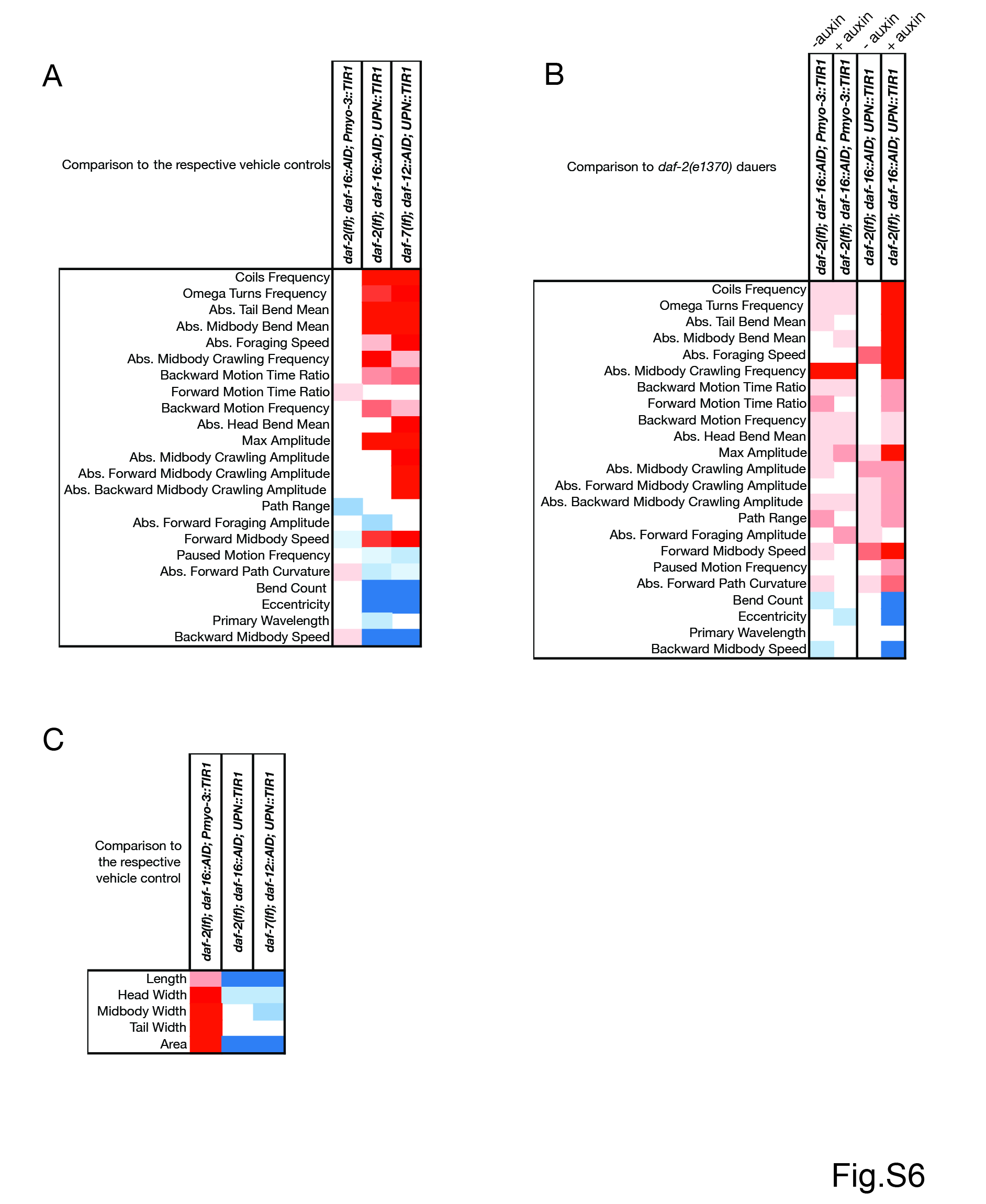

Supplement: S6 Fig — (A) Locomotory features of dauers with DAF-16 depleted from neurons and body wall muscle and DAF-12 depleted from neurons, as compared to their respective vehicle controls. (B) Locomotory features of dauers with DAF-16 depleted from neurons and body wall muscle, as compared to daf-2(e1370) control dauers. (C) Effect of DAF-16 depletion from body wall muscle on dauer morphology. The data underlying this figure can be found in S2 Data. (TIF) [file pbio.3001204.s006.tif]

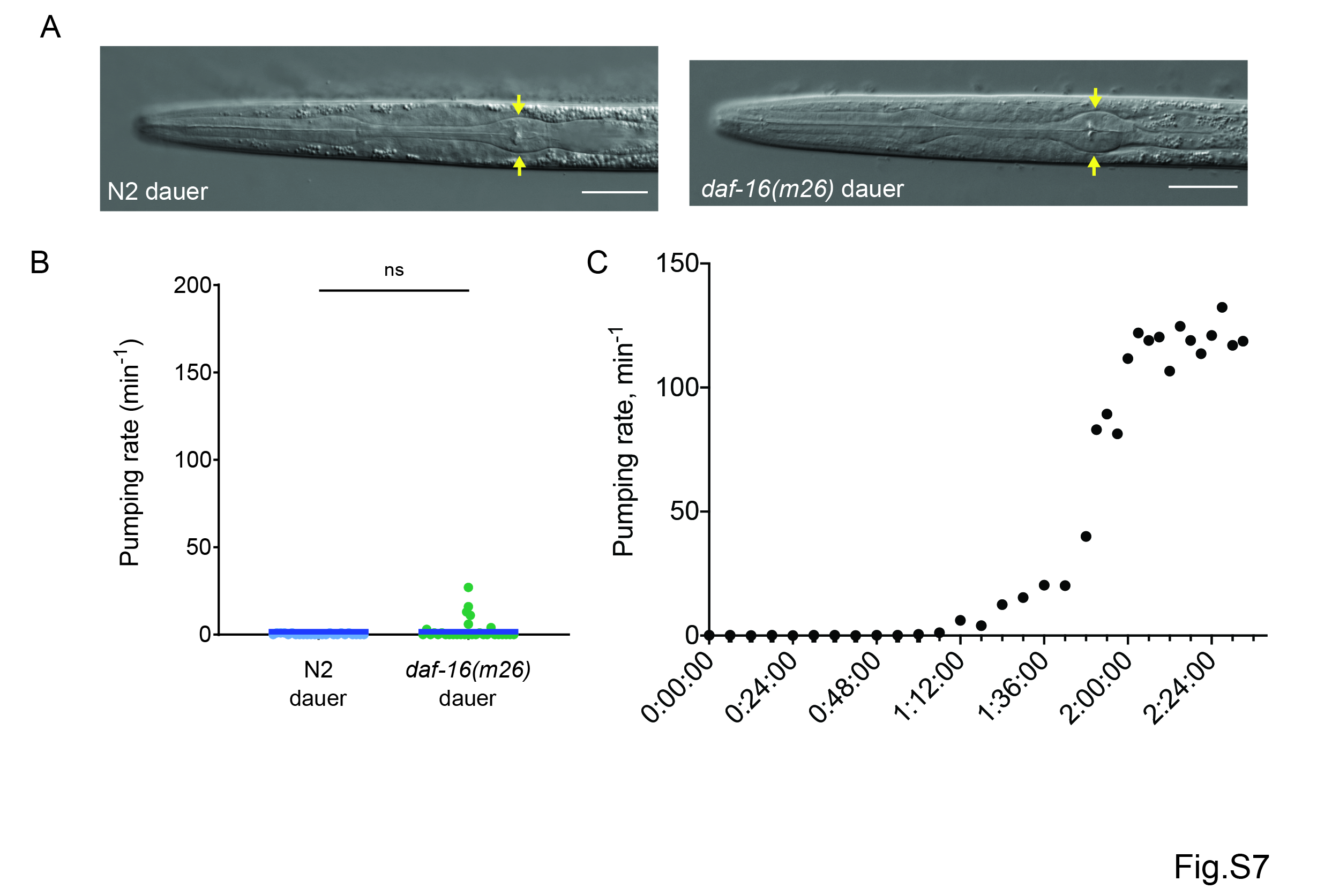

Supplement: S7 Fig — (A) Pharyngeal morphology in wild-type (N2) and daf-16(m26) starvation-induced dauers. Yellow arrows show width of the terminal bulb of the pharynx. Scale bars, 20 μm. (B) Pharyngeal pumping rate in N2 and daf-16(m26) starvation-induced dauer animals. Blue horizontal line represents median for ≥25 animals per genotype. ns indicates p = 0.36 in 2-tailed Mann–Whitney test. (C) Pharyngeal pumping rate in wild-type animals recovering from starvation-induced dauer stage. The data underlying this figure can be found in S1 Data. (TIF) [file pbio.3001204.s007.tif]
