# Supplementary material for: DAF-16/FoxO and DAF-12/VDR control cellular plasticity both cell-autonomously and via interorgan signaling
Source: PLoS Biol. 2021 Apr 23;19(4):e3001204. doi: 10.1371/journal.pbio.3001204 (PMC8099054; doi:10.1371/journal.pbio.3001204)
Supplement: Attachment — Submitted filename: Response to Reviewers comments v2.docx [file pbio.3001204.s010.docx]

**RESPONSE TO REVIEWER** *(our responses in red)*

**Reviewer #1**: The C. elegans dauer larva is an excellent model for studying the molecular basis for phenotypic plasticity. Molecular pathways that govern dauer formation are evolutionarily conserved, suggesting that mechanisms underlying phenotypic plasticity in C. elegans dauers will inform studies on phenotypic plasticity in other organisms. While the logic of insulin-like, TGFbeta-like, and hormonal signaling pathways that control dauer entry and commitment is reasonably well understood, how the three major transcription factor targets of these pathways function to execute the dauer developmental fate is largely unknown. In this manuscript the authors use state-of-the-art reagents they have developed to perform a detailed analysis of the spatiotemporal requirements for dauer-specific phenotypic plasticity. The study is enabled by the generation of strains that are tagged at endogenous transcription factor loci with fluorescent proteins and an auxin-inducible degron (AID). These strains permit spatiotemporal control of gene expression while obviating caveats of previous studies using transgenic constructs that overexpress genes and may lack regulatory elements that control endogenous gene expression.

The experiments presented define sites and times of action for DAF-3/SMAD, DAF-16/FoxO, and DAF-12/VDR in the execution of various aspects of the dauer developmental program. An enormous amount of data is presented. The experiments are well-executed, and the data are convincing. Taken together, the data provide new insights into temporal, cell-autonomous, and cell-nonautonomous actions of DAF-16/FoxO and DAF-12/VDR in dauer execution, confirm previous studies on the nonautonomy of TGFbeta and insulin-like signaling in dauer regulation, and resolve conflicting data in the literature regarding sites of action of DAF-16/FoxO in dauer regulation. The paper will be of great interest and utility to dauer mavens as well as developmental biologists, cancer biologists, and anyone with an interest in phenotypic plasticity.

I have a few minor comments:

1. Fig. 12B: in light of the unexpected result that DAF-2DN expression in the pharynx does not induce nuclear translocation of DAF-16 in the pharynx, it would be nice to know the extent to which ubiquitous overexpression of DAF-2DN induces dauer arrest in wild type animals at 25C. My guess is that even in the context of DAF-2DN overexpression, a low level of wild-type DAF-2 dimers form and induce DAF-16 phosphorylation and cytoplasmic retention. This is not absolutely necessary but could be included if the authors have this data in hand.

*We appreciate Reviewer #1’s generous comments and are pleased that our manuscript is deemed of interest for a broad audience. Regarding ubiquitous overexpression of DAF-2DN, we know that ubiquitous complete loss of activity of daf-2 results in embryonic lethality (daf-2 null is not viable). Hence, even if we try to obtain a line with an extrachromosomal array for ubiquitously expressed DAF-2DN, we will only be able to make a line if the dominant negative effect is incomplete, i.e., some residual activity of wild-type DAF-2 dimers remains. Since the system we use is not inducible, we will be selecting for lines that do not produce constitutive dauers and have lower DAF-2DN expression. Therefore, we have only used tissue-specific DAF-2DN lines in our experiments. Arguably, with an inducible system it might be possible to address this question by inducing DAF-2DN before commitment to reproductive development in early L2, for example.*

2. Typo in Fig. S2: "intestinal"

*Fixed.*

3. Fig. S4: "mgDf50"

*Fixed.*

**Reviewer #2**: The manuscript by Hobert and colleagues investigates longstanding questions about the site of action and timing of regulatory pathways involved in the initiation and maintenance of the dauer pathway of C. elegans. Dauer formation is a remarkable instance of developmental plasticity in nematodes, but in addition, the genetic analysis of dauer entry of C. elegans has served as a paradigm for understanding the role of evolutionarily conserved neuroendocrine signaling pathways in organismal physiology. This important study addresses longstanding questions in the field through the incisive application of CRISPR-based-methods that represent a marked improvement over prior analyses, and the findings define how cell-non-autonomous signaling can coordinate organismal responses across tissues.

The generation and analysis of strains carrying in-genome tags to monitor expression revealed DAF-3 expression to be increased with starvation. DAF-16 is shown to undergo nuclear localization in the intestine, but not in the neurons upon food deprivation, revising and adding to prior studies of DAF-16 localization in response to stress, but here with a more rigorous and solid experimental foundation. DAF-12 expression is also analyzed. Further engineering of auxin-inducible degron tags, coupled with tissue-specific expression of the TIR1-interaction domain, enable the authors to conduct essentially targeted mosaic-like analysis in a cell-type specific manner with temporal control. DAF-3 is noted to function in the nervous system, and somewhat surprisingly in the ciliated neurons given the prior report of Ashrafi and colleagues (Greer et al., 2008) reporting evidence that DAF-1 functions in RIM/RIC interneurons. This may merit some discussion.

*We are thankful to this reviewer for pointing out the reference on DAF-1. We now mention this matter in the legend to Fig.4A. The simplest explanation for this apparent discrepancy is that Greer at al. test for sufficiency (i.e. where daf-1 is able to rescue if misexpressed in a mutant background), while we test for necessity (i.e., where daf-3 removal is required, through removal of the gene). In light of our results, one could have expected that their daf-1 expression with osm-6 or bbs-1 promoter may also rescue the phenotype, but again, this is a sufficiency experiment and hence not quite comparable. Also, as panneuronal driver we use ift-20, not osm-6 or bbs-1. This is because we found long ago that the osm-6 driver does not provide as uniform sensory neuron expression as ift-20 does. Lastly, the conclusion of the daf-1 experiments is similar to that of our daf-3 experiments in the sense that the TGFb system acts cell-autonomously.*

The DAF-16 discussion is delicately handled but provides very interesting and again, more solidly grounded (in terms of methodology, there are fewer caveats) data that will be extremely helpful for the many investigators studying DAF-16 in diverse aspects of C. elegans physiology. The authors carefully note that tagging of the genes involved do not grossly affect function in the dauer entry phenotypes/suppression are left unaffected, but the reduced lifespan of daf-2(e1370);daf-16::AID relative to daf-2 (Fig 10) that is noted by the authors may merit some further discussion, as these data suggest some degree of reduction of function of DAF-16 from the tagging. Importantly, I do not think this caveat affects the major conclusions from the experiments using daf-16::AID in the manuscript, as these experiments are all internally controlled, and if anything, the effects observed are likely to be relatively smaller if the tagging affects baseline activity.

*Yes, correct, we mention this on page 16 and have now further added a sentence + reference that points out that previous work has shown that the role of daf-16 in ageing is very dosage sensitive (Bansal et al. 2014). We note that we do not observe such hypomorphic effects for the dauer phenotypes.*

The authors are also able to simply and elegantly define the roles of these pathways in not only initiation, but maintenance of the dauer state, though the differing kinetics of recovery may merit some discussion. For this reviewer, these data would be plenty enough for a highly impactful study.

*Yes, we are excited that we were able to implicate all three pathways in maintaining the dauer stage, as shown by our conditional protein removal and ensuing dauer recovery. However, we are a little reluctant to read too much into the differences in recovery rate at this point. Those may not necessarily relate to different functions of the pathways, but may relate more to technical issues and/or protein levels.*

But the authors go on to use these strains to define, in rigorous and elegant detail, all of the findings that are basically summarized in the table in Figure 14. Far from simply being a listing of phenotypes that are modified by cell-autonomous vs. cell-non-autonomous activities, these data begin to put together a picture of organismal physiology around dauer initiation, entry, and maintenance, decoupling some aspects specific to individual pathways from the overall phenotype, and underscoring the manner in which neuronal signaling can control organismal physiology. There are a few points that I would suggest might benefit from further discussion, and Figure 2A has a typo ("instestinal nuclei" next to the purple circles), but nothing that takes away substantively from this beautifully conceived, rigorously executed, and clearly presented story.

*Typo in Figure 2A has been corrected.*

**Reviewer #3**: This is a masterful and complete genetic characterization of how the global transcriptional output of insulin, TGF beta, and the daf-12 NHR ligands remodel a growing reproducing animal into the very different dauer animal of C. elegans. The experiments are complex but very well explained, and the data presentation is very clear. It is written with a focus on neural activity that subserves the changes from dauer to non dauer life cycles. And it is written in a manner that allows even non experts in C. elegans to follow the logic. The literature on where insulin signals for dauer arrest vs reproductive development has been conflicting because of transgene issues and this paper clears it all up. This is a large field in C. elegans and so there will be many interested readers. But my one suggestion to the authors is to add a paragraph (am I kidding? This paper is already super long) about how the circuits of neural response to insulin signaling defects are likely to present in the millions of insect species that undergo diapause and try to disperse to new niches. Also, dauers are the dispersal stage of C. elegans, hitching a ride onto insects to fly to new ecosystems when the current niche begins to suck. Dispersal stages of insects probably use ancestrally related pathways, including the neurons that this paper highlights. This paper does not have prove that the circuits are present in insects but they can discuss the possibility.

*We are very grateful to the reviewer for prodding us into the broadening the horizon a little, in terms of considering other species. We have expanded the last paragraph of the Discussion to discuss that a combo of insulin and steroid-hormonal systems also control diapause in a number of different systems. Apart from insects, the most notable comparison is killifish, which undergoes diapause in a manner that also involves insulins and vitamin D receptor. In addition, we have added a new paragraph at the end of the discussion that goes into the issue of dispersal behavior that the reviewer brings up.*

But the paper needs no additional experiments. Read my lips Reviewers 2 and 3: No additional experiments. There are a ton of great experiments in the many figures. Very impressive.

But many of the Figures can become Supplementary Figures

Here are the Figures that I suggest become Supplementary

Figure 1

Figure 3AC

Figure 4 fuse to Figure 3 BD

Figure 7

Figure 8

Figure 10

Figure 12

I would not suggest these moves except that a 14 Main Figure paper is longer than any I have ever seen. The self assurance of the authors is impressive.

Congratulations to the authors on producing such a wonderful paper.

As these figures make important points, we would be averse to see them downgraded to only Supplementary Figures and prefer to keep the figures organized as is. One of the reasons we submitted this paper to PloS Biology was that there is no limit to figure number.

**Reviewer #4**: The manuscript describes a set of new tools to assess the temporal and tissue-specific requirement of daf-3, daf-16, daf-12, three major transcription factors in regulating dauer formation in C. elegans. In general, the study is well-designed and well executed. The data presented are rigorous and the conclusions drawn are appropriate.

The manuscript is fairly complex to get through, because a lot of details were being presented. Figure 14 certainly helps, but perhaps additional summary figure can be added in some of the earlier figures. e.g. the various consequences of neuronal depletion of daf-16 and daf-12 can be compared and summarized earlier. Then another summary figure can be used for the pharyngeal phenotypes. Further, the idea presented in Figure 13 deserves an additional model figure to illustrate the bigger picture model about collaboration between DAF-16 and terminal selector TFs.

*We are thankful to this reviewer for the figure suggestions, and we have added a conceptual schematic into the summar Figure 14 to explain these collaborations.*

Also, regarding to overall dauer decision, the data seem to suggest that elimination of DAF-16 in a single tissue is not sufficient to block the dauer program. **It follows then that whether the authors have tried removal of daf-12 or daf-16 in more than one tissue? e.g. neuron combined with intestine, etc?**

*We have tried removing DAF-16 from neurons and intestine in the same animals and did not observe suppression of dauer formation. We chose not to include these data in the manuscript, because of the lack of other tissue combinations. At some point, a combination of tissues ought to recapitulate ubiquitous protein depletion but we in our experiments had not reached that point. No experiments with combined tissue-specific depletion of DAF-12 have been conducted.*
